# Recyclability of Zinc Palmitate-Based Composites in Fatty Acid Methyl Ester Production from Oil Feedstocks at Varied Acidity

**DOI:** 10.1021/acsomega.4c02740

**Published:** 2024-10-07

**Authors:** Charoen Posa, Palmpapat Junpuek, Warisara Woranuch, Kittichai Chaiseeda, Kornkanya Pratumyot, Nongnuch Sungayuth, Siwaporn Meejoo Smith

**Affiliations:** † Center of Sustainable Energy and Green Materials, Faculty of Science, Mahidol University, 999 Phuttamonthon Sai 4 Rd., Salaya, Nakhon Pathom 73170, Thailand; ‡ Department of Chemistry, Faculty of Science, King Mongkut’s University of Technology Thonburi, 126 Pracha Uthit Rd., Bang Mod, Thung Khru, Bangkok 10140, Thailand; § Organic Synthesis, Electrochemistry and Natural Product Research Unit (OSEN), Department of Chemistry, Faculty of Science, King Mongkut’s University of Technology Thonburi, 126 Pracha Uthit Rd., Bang Mod, Thung Khru, Bangkok 10140, Thailand; ∥ Supramolecular Chemistry Research Unit, Department of Chemistry, Faculty of Science, King Mongkut’s University of Technology Thonburi, Pracha Uthit Road, Bang Mod, Thung Khru, Bangkok 10140, Thailand; ⊥ School of Interdisciplinary Study, Mahidol University Kanchanaburi Campus, 199, Lumsum, Saiyok District, Kanchanaburi 71150, Thailand; # Department of Chemistry, Faculty of Science, Mahidol University, 272 Thanon Rama VI, Thung Phaya Thai, Ratchathewi, Bangkok 10400, Thailand

## Abstract

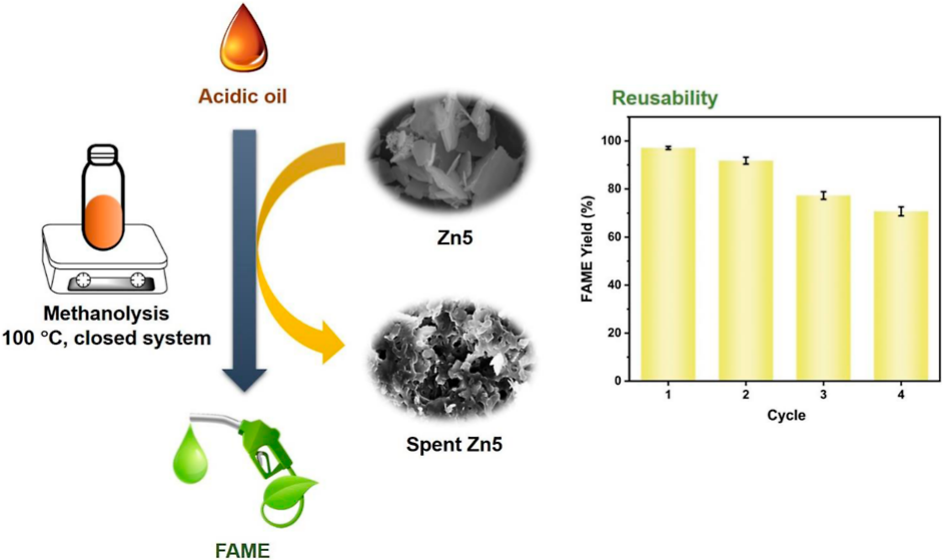

Zinc hydroxide nitrate (Zn5) is one of the layered metal hydroxide materials, which has been extensively reported as an effective bifunctional catalyst for biodiesel production from acidic oils. This report gives a comprehensive summary of the reactivity of Zn5 in the methanolysis of various oil feedstocks, plant oils, free fatty acids, and other acidic oils. Notably, as evidenced by this work, Zn5 is highly effective in converting acidic oils [palmitic acid/palm oil (PO)] to fatty acid methyl ester (FAME or biodiesel) at high yields ranging from 80 to 95%, withstanding acid content up to 10% without soap formation. The high FAME yields result from complex methanolysis and hydrolysis processes, e.g., transesterification of triglycerides in the PO, esterification of palmitic acid, and hydrolysis of the triglycerides. Despite this, Zn5 is nonrecyclable because it is unstable in the reaction media and transforms into zinc hydroxide nitrate/zinc palmitate (Zn5/ZnP) composites. The Zn5/ZnP composites were suitable for use in FAME production from PO at 100 °C for 2 h by using a methanol-to-oil molar ratio of 30:1, yielding high FAME yields of 97 and 70.7% in the first and fourth cycles, respectively. This study added better insight into how to effectively produce FAME from oil feedstocks of varying acidity by using zinc layered hydroxide- or zinc carboxylate-based materials.

## 1 Introduction

Alcoholysis of oil or fat produces fatty acid alkyl esters, which are commonly referred to as biodiesel, due to their chemical compositions being similar to petrodiesel. Methanol is frequently used to reduce the cost of the methanolysis products known as fatty acid methyl esters (FAME). The terms “transesterification of vegetable oils” and “esterification of free fatty acids (FFAs)” are often used to describe alcoholysis related to biodiesel production. Although homogeneous catalysts (e.g., H_2_SO_4_, KOH, or HCl) produce high yields of biodiesel at high rates,^1,2^ heterogeneous catalysts (solid catalysts) are frequently favored because they address the shortcomings of homogeneous catalysts, such as corrosiveness including limited selectivity and recovery.^3,4^

Zinc layered hydroxide with the general formula of (Zn^2+^)_*x*_(OH^–^)_2*x*−*my*_(A^*m*–^)_*y*_·*n*H_2_O, where A^*m*–^ represents anions stabilized between zinc hydroxide layers, was previously reported as an effective bifunctional heterogeneous catalyst, containing both acidic and base active sites, in biodiesel production.^5^ For example, zinc hydroxide nitrate (Zn_5_(OH)_8_(NO_3_)_2_·2H_2_O or Zn5) was used in FAME production from the reactions between either lauric acid or palm oil (PO) and hydrate ethanol.^6,7^ It was claimed that the reactions involved in the conversion of lauric acid and PO to fatty acid ethyl ester (FAEE) are due to esterification and transesterification, respectively. Nevertheless, according to a more recent publication,^8^ the high yields of FAEE produced from PO and hydrate ethanol could be due to the combination of transesterification and hydrolysis of triglycerides in PO, followed by esterification of the hydrolytic byproducts (FFAs). It was addressed that high FAME yields from methanolysis of acidic oils were due to the simultaneous transesterification of triglycerides in the oil and esterification of acidic feedstock (oleic acid containing soybean oil) together with hydrolysis of triglycerides as side reactions. Both acid and basic sites on Zn5 are responsible for these chemical transformations. Another study used thermally treated Zn5 materials to achieve high FAME yields from the methanolysis (or transesterification) of castor oil or triacetin. These papers^6−8^ did not provide specific information on the number of times the Zn5 or the thermally treated Zn5 was recycled or reused. The study’s primary goal was to demonstrate the catalyst’s efficacy in the esterification of FFAs and the transesterification of vegetable oils in a one-time application. No detailed insights into the recyclability in terms of the number of cycles for biodiesel production were summarized. Furthermore, zinc hydroxide acetate (Zn–Ac) and Zn5 materials were applied as catalysts for the synthesis of biodiesel from soybean oil.^9^ The Zn5 material, produced via precipitation in a basic aqueous solution, displayed stability over three cycle tests, indicating its potential for reuse in biodiesel synthesis processes. However, Zn–Ac was found to be unstable, as it transformed into Zn glycerolate in the reaction medium, implying that it may not be effectively recyclable for repeated use in similar processes. As a result, only one available report^9^ highlights the recyclability of Zn5 material in biodiesel production from the transesterification of triglycerides and methanol. In this investigation, Zn5 was reexamined whether it is applicable as a catalyst in biodiesel production from PO and palmitic acid-containing palm oils (simulated acidic oil feedstocks) in multiple runs. The efficiency and recyclability limits of Zn5 are described in detail based on experimental evidence. Multiple-cycle biodiesel production from various oil feedstocks (at varying acidities) and methanol was further studied to explore the feasibility of Zn5 at expanded limits. The relationships between the acidity of oil feedstock, the characteristics of spent Zn5, and the FAME yields are discussed and compared with those in previous reports. This research should help establish a recommended protocol for the utilization of Zn5 or other metal-layer hydroxide materials in biodiesel production.

**Figure 1:**
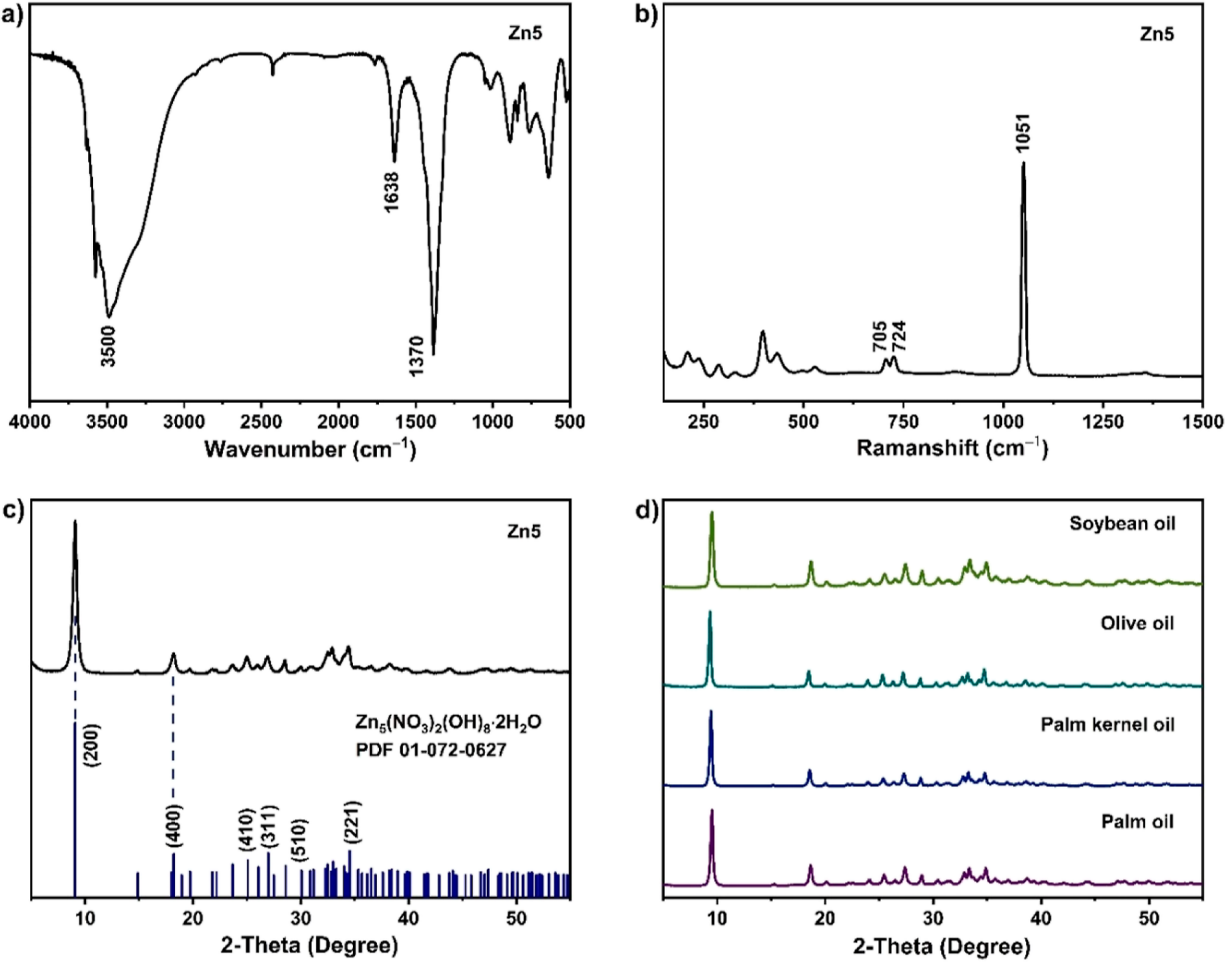
(a) FTIR spectrum, (b) Raman spectrum, and (c) XRD profile of the Zn5 material, including (d) XRD patterns of the spent Zn5 after the methanolysis of various plant oils (soybean, olive, palm kernel, and palm oils).

**Table 1:** Screening Test, FAME Yields Obtained from Methanolysis of Various Plant Oil or Free Fatty Acid Feedstocks^a^

Feedstock			Reaction conditions	% FAME yield
Type	Name	Acidity (mg NaOH/g)		
plant oil	olive oil	10.68 ± 0.29	3% wt Zn5	57.86 ± 1.54
	soybean oil	10.25 ± 0.06		18.84 ± 2.03
	palm oil	10.56 ± 0.05	MeOH/oil = 30:1	11.73 ± 1.27
	palm kernel oil	10.35 ± 0.07		9.39 ± 3.41
FFA	stearic acid	93.66 ± 0.21	100 °C, 2 h	81.67 ± 1.54
	lauric acid	134.53 ± 0.49		60.19 ± 2.61
	palmitic acid	98.10 ± 4.55		59.20 ± 1.15
	oleic acid	81.72 ± 0.25		45.77 ± 1.93

a Reaction conditions were chosen similar to those reported by Smith et al.,^8^ 2023, to compare FAME yields obtained from similar reactor type. The acidity of feedstock was determined by a NaOH titration method. Yields of 80% and above are sufficient to declare effective FAME production from methanolysis, and further tuning of the reaction parameter could increase the FAME yields even further.

**Figure 2:**
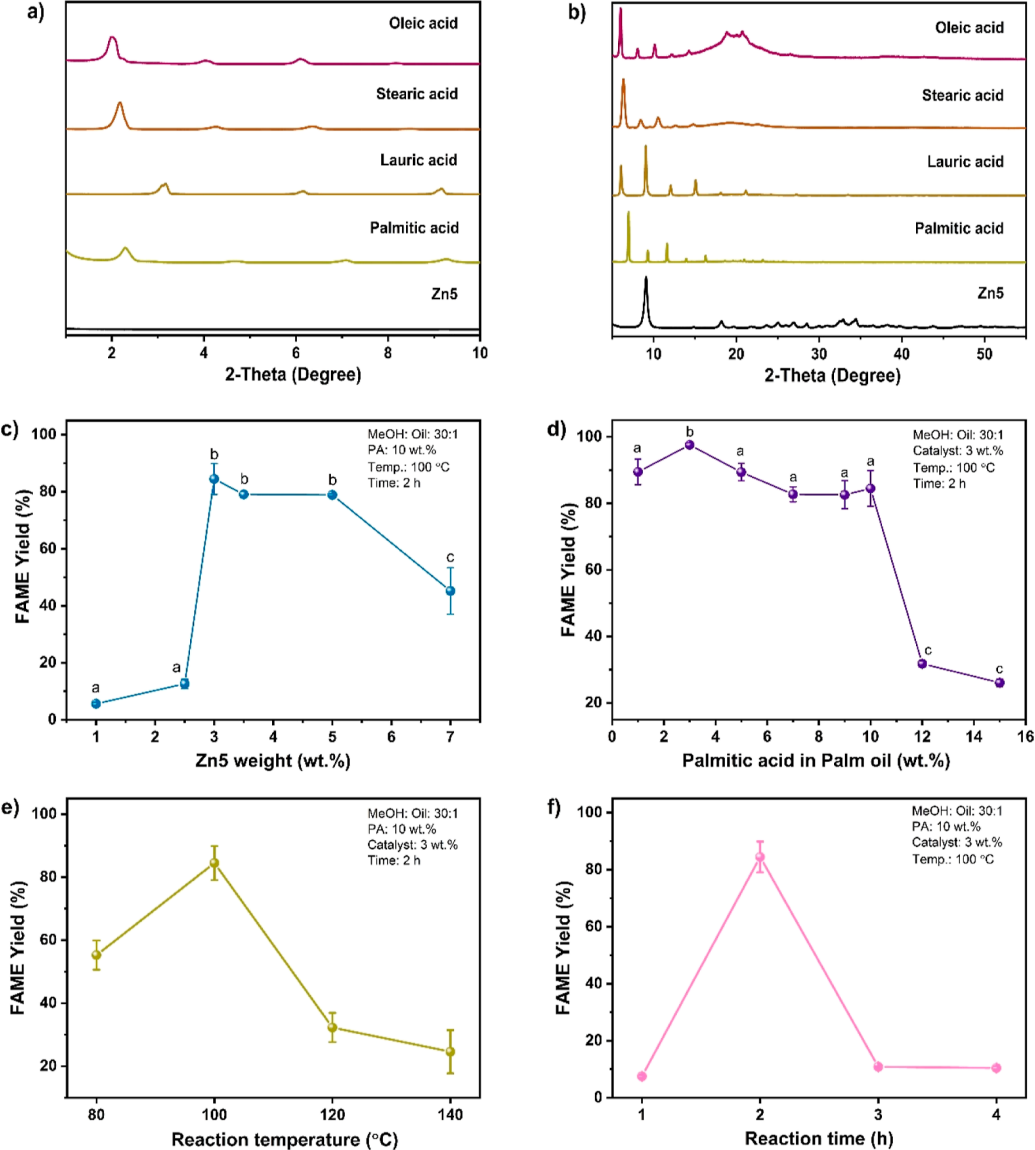
(a) Low-angle and (b) high-angle XRD patterns of the spent Zn5 after the methanolysis of various FFAs (oleic, stearic, lauric, and palmitic acids) compared to that of the as-prepared Zn5 material; effects of (c) Zn5 loadings, (d) % wt palmitic acid, (e) reaction temperature, and (f) reaction time on FAME yields.

**Table 2:** Screening Test, FAME Yields Obtained from Methanolysis of Acidic Feedstocks; (1) Acidic Oils Prepared in This Study by Mixing a Plant Oil and a Small Amount of FFA^a^^,^^b^

feedstock composition		feedstock, acidity (mg NaOH/g)	reaction conditions	% FAME yield
10% wt FFA	plant oil			
palmitic acid	palm oil	16.60 ± 0.07	3% wt Zn5	83.82 ± 2.97
lauric acid		18.81 ± 0.15		77.98 ± 3.14
stearic acid		13.79 ± 0.32		32.63 ± 0.14
oleic acid		12.92 ± 0.92	MeOH/oil = 30:1	9.47 ± 0.47
palmitic acid	palm kernel oil	10.35 ± 0.07		9.52 ± 1.66
	olive oil	10.68 ± 0.29	100 °C, 2 h	7.85 ± 1.10
	soybean oil	10.25 ± 0.06		5.46 ± 0.20

a The highest FAME yields were achievable from palmitic acid/PO mixtures, with 10% wt palmitic acid. Additional results in Table 2 imply that appropriate matchings between FFA and plant oil types are required for high FAME yields as very low FAME yields were obtained when palmitic acid was mixed with other types of plant oils.

b Yields of 80% and above are sufficient to declare effective FAME production from methanolysis, and further tuning of the reaction parameter could increase the FAME yields even further.

**Figure 3:**
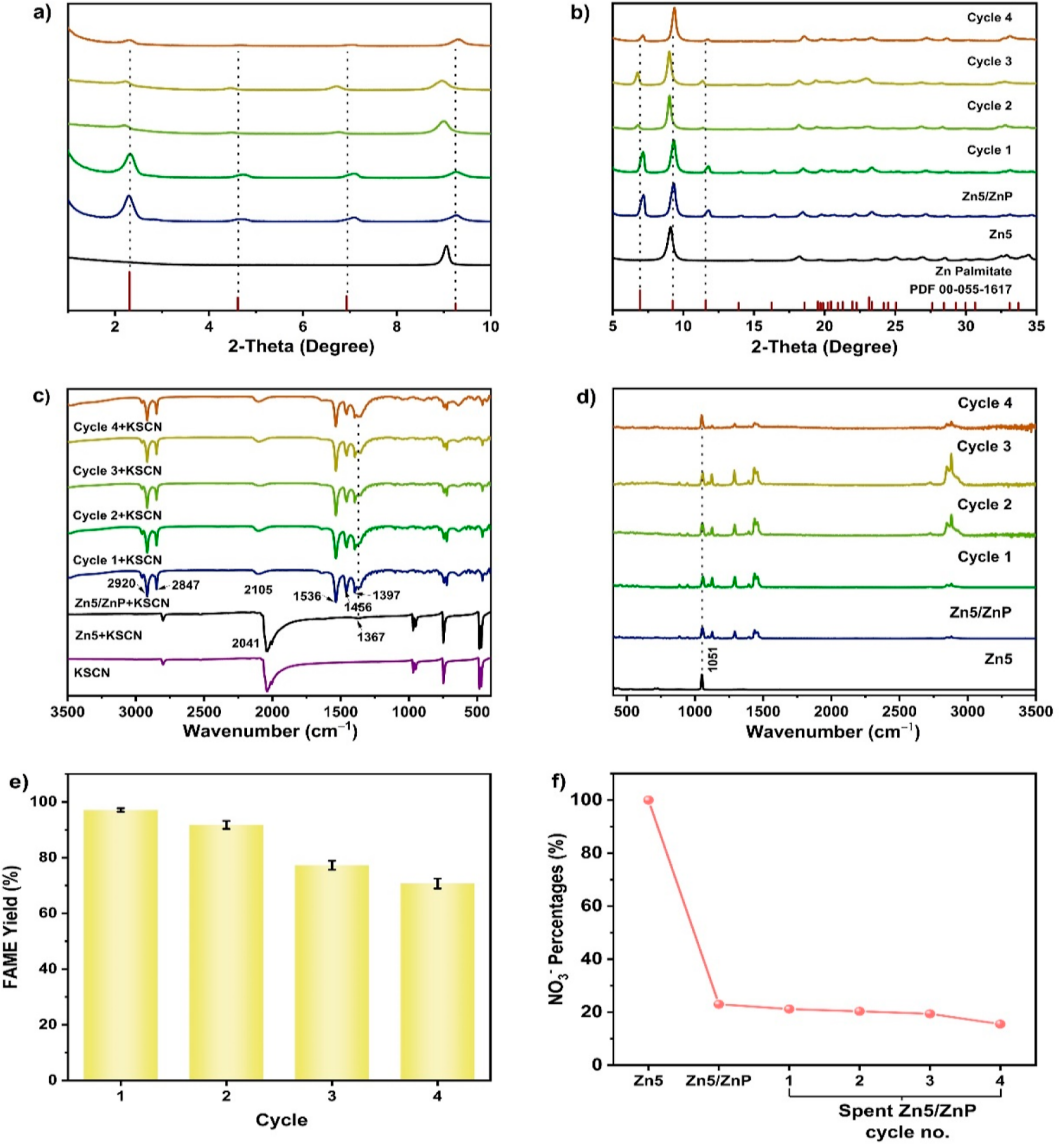
(a) Recyclability of Zn5/ZnP in FAME production from PO and methanol and (b) nitrate content in the Zn5-based materials according to IR data; (c) IR spectra and (d) Raman spectra for Zn5/ZnP, spent Zn5/ZnP compared with Zn5; (e) recyclability of Zn5/ZnP in FAME production from PO and methanol; and (f) nitrate content in the Zn5-based materials according to IR data. The spent Zn5/ZnP was obtained from methanolysis of PO using 3% wt of Zn5/ZnP, a MeOH to oil ratio of 30:1, at 100 °C for 2 h.

**Figure 4:**
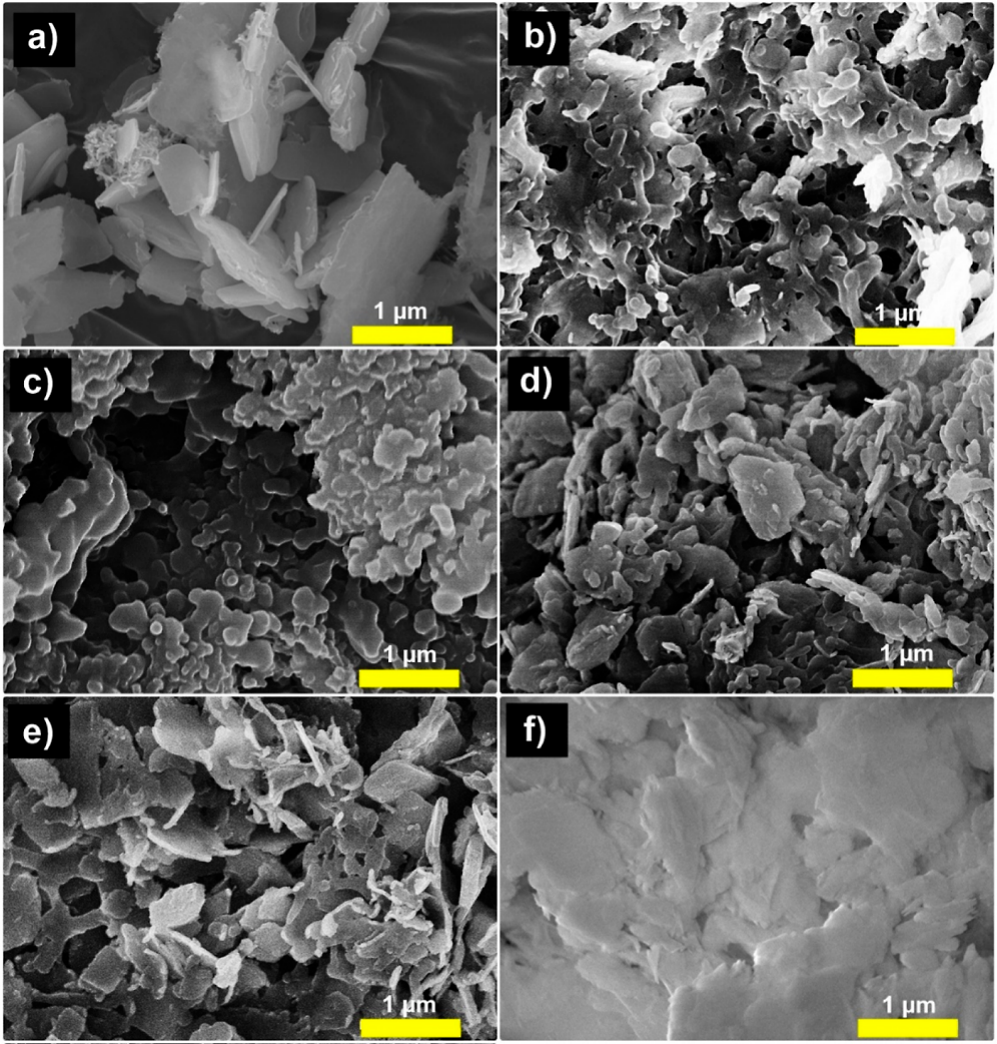
SEM images of (a) Zn5, (b) Zn5/ZnP, as well as the spent Zn5/ZnP (c) cycle 1, (d) cycle 2, (e) cycle 3, and (f) cycle 4 of FAME production from 3PA/PO feedstocks.

## 2 Materials and Methods

Zinc nitrate hexahydrate (Zn(NO_3_)_2_·6H_2_O, AR grade) and sodium hydroxide (NaOH, pellet, AR grade) were supplied by KEMAUS and Central Drug House (P) Ltd., respectively. Methanol (Honeywell, AR grade) and ethanol (AR grade, KEMAUS, Australia) were used without purification. FFAs employed in this study include oleic acid (pharma grade, Panreac, USA), lauric acid (AR grade, QReC, New Zealand), stearic acid (AR grade, Chemi Science Industry Co., Ltd., Thailand), and palmitic acid (AR grade, Scharlau, Spain). Oil feedstocks PO (food grade, OGA Band, Suksomboon vegetable oil company, Thailand), palm kernel oil (food grade, Flower brand, Patum vegetable oil company, Thailand), soybean oil (food grade), and olive oil (food grade, KC brand, Krungthepchemi Co., Ltd., Thailand) were purchased from a local shop in Thailand. In this study, *d*–Chloroform (AR grade, CDCl_3_–0.05% v/v TMS–silver foil, Cambridge Isotope Laboratories) was utilized as a NMR solvent. Other solvents were methanol and tetrahydrofuran (THF, HPLC grade, QReC, New Zealand). Chemicals used for titration experiments were sodium chloride (AR grade, Loba Chemie, India), benzoic acid (AR grade, Merck, Germany), phenolphthalein (AR grade, KEMAUS, Australia), and methyl red (indicator grade, QReC, New Zealand). Zinc hydroxide nitrate (Zn5) was prepared through a precipitation method under a basic condition, following previous reports.^10^

### 2.1 Sample Characterization

The AOAC official method 940.28^11^ was employed to determine the acidity of feedstocks (plant oils, FFA, or acidic oils) by using a NaOH titration method and phenolphthalein (1% wt by volume of ethanol) as an indicator.^12,13^ Briefly, any acid in 250 mL of ethanol was neutralized by 0.1 N NaOH(aq) with the addition of 3–4 drops of ethanolic phenolphthalein solution. After the solution turned from colorless to light pink, 10 g of an oil (or FFA or acidic oil) was poured into the solution, and the mixture was stirred at 60 °C for 20 min, followed by vigorous hand shaking for 1 min to obtain a well-mixed sample. Three to four drops of ethanolic phenolphthalein solution were added into the above mixture, which was subjected to titration with 0.1 N NaOH(aq). The volume of NaOH(aq) required to turn the color of the mixture from colorless to light pink was utilized to calculate the acidity of feedstocks by using the given equation.
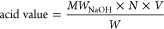
where *MW*_NaOH_ is the molecular weight of NaOH (40.0 g/mol), *N* is the normality of sodium hydroxide solution (0.1 N), *V* is the volume of sodium hydroxide used (mL), and *W* is the oil sample (g).

The acidity of Zn-based materials was determined using a NaOH titration method. In detail, a suspension of 50 mg in 15 mL of 0.4 M NaCl(aq) with the addition of 3–4 drop of ethanolic phenolphthalein solution was stirred at room temperature for 12 h. Then, the suspension was filtered, and the colorless filtrate was collected and its volume adjusted to 15 mL by deionized water. The filtrate solution was titrated with 0.1 N NaOH(aq), and the acidity of the Zn-based material was derived by the following equation.
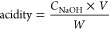
where *C*_NaOH_ is the concentration of the sodium hydroxide solution (0.02 M), *V* is the volume of sodium hydroxide used (mL), and *W* is the weight of solid sample (g).

To obtain the basicity of Zn-based materials, aqueous methyl red (1% wt by volume of ethanol, 3–4 drops) was added to a mixture of 50 mg of solid material dispersed in 15 mL of 0.4 M NaCl(aq). After being stirred at room temperature for 12 h, the mixture underwent filtration, and the volume of the filtrate was adjusted by water to 15 mL. The colorless solution was then titrated with 0.02 M benzoic acid (aq). The volume of acid required to make a pale orange solution was applied in the equation below to calculate the basicity of the Zn-based material.
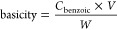
where *C*_benzoic_ is the concentration of the benzoic acid solution (0.02 M), *V* is the volume of benzoic acid used (mL), and *W* is the weight of the solid sample (g).

The characteristics of all solid samples were analyzed by various techniques. Powder X-ray diffraction (Bruker-D2 PHASER equipped with a Cu Kα, USA) and SEM (JEOL-FESEM, JSM-IT500, Japan) were employed to gain information on crystal structure and morphology, respectively. Diffraction profiles were recorded at 40 kV and 35 mA in the 2-theta range of 2–50°. SEM analysis was performed at 10 kV to record SEM images at a magnification of 25,000×. Results from IR (PerkinElmer-Frontier, Waltham, MA, USA; 400–4000 cm^–1^) and Raman spectroscopy (HORIBA scientific—XploRA PLUS Raman Microscope, Japan; exciting wavelength of 785 nm) were used to identify the chemical composition of the samples.

Additionally, the IR spectra of Zn5 and the spent Zn5 were measured by adding potassium thiocyanate (KSCN) as an internal standard. An accurately weighed amount of 2.5 mg of KSCN was added to 30.0 mg of Zn5 (or spent Zn5). The mixture was grounded and homogenized by agate mortar. The FTIR spectrum of mixtures was recorded in the range from 4000 to 400 cm^–1^. Typically, KSCN gives an absorption peak at 2041 cm^–1^, which corresponded to S–CN stretching, while the IR signal of nitrate groups was observed at 1367 cm^–1^ (N–O stretching). The peak areas of those two IR peaks were derived by peak integration analysis using the origin software (Origin, LabPro-Origin 2021, Northampton, MA, USA). The nitrate content was evaluated with respect to the number of moles of KSCN internal standard by using eqs 1–3.
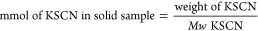
1
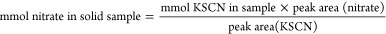
2
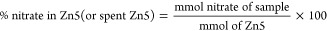
3

### 2.2 Methanolysis and Determination of FAME Yields

Various feedstocks were employed in this study, including plant oils, FFAs, and acidic oil feedstocks, which are mixtures of specific plant oils and fatty acids. For each feedstock, methanolysis was performed in an ace-pressured tube equipped with a Teflon screw closure lid using a closed reactor system equipped with a magnetic stirrer and an oil bath. Controlled methanolysis parameters are the reaction temperature and time, catalyst loading, and the methanol-to-oil molar ratio. For screening tests, a suspension of 150 mg of Zn5 in 7.2 mL of methanol was heated to 100 °C for 5 min. Then, an oil feedstock was added into the suspension, with a methanol-to-oil ratio of 30:1 and at 3 wt % Zn5 by weight of the oil. The methanolysis reaction mixture was kept at 100 °C under a stirring condition for 2 h. After that, the mixture was cooled at room temperature until three layers^14^ in the ace-pressured tube were observed. Fifty microliter of the top layer was drawn out by a Pasteur pipet and dissolved in 1 mL of d-chloroform, and this solution was subjected to ^1^H NMR analysis on a Bruker Avance 400 NMR spectrometer. Each ^1^H NMR spectrum was analyzed to obtain the % FAME yield from the methanolysis reaction, by using eq 4(13,15)
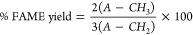
4where *A–CH*_3_ and *A–CH*_2_ are the areas of NMR peaks corresponding to methyl esters (3.65 ppm) and methylene protons (2.30 ppm), respectively. The multiplication numbers, 2 and 3, are applied based on the two protons in methylene carbons in triglycerides and three protons in methyl carbons in methyl esters, respectively.

Later, PO was used as a representative of low-acidity oil feedstock, whereas series of acidic oil feedstock samples were prepared by mixing palmitic acid (denoted as PA) at varying contents (2–20% wt) with PO. The acidic oil feedstock samples were denoted as *n*PA/PO, in which “*n*” represents the percent of palmitic acid by weight with respect to the weight of PO. Methanolysis of *n*PA/PO samples was conducted following the method described earlier, by varying reaction temperatures (80–140 °C), reaction times (1–4 h), and Zn5 loadings (1–7% wt). FAME yields were determined by ^1^H NMR spectrum analysis as explained above. The results gave optimum methanolysis reaction conditions for low-acidity oil and acidic oil.

### 2.3 Recyclability Test

Multiple-run methanolysis of PO (or acidic oils) was carried out using the optimum reaction parameters previously acquired, while FAME yields were estimated using the previously described protocol. The spent Zn5 was recovered by conducting 15 min centrifugation (Hettich ZENTRIFUGEN, D-7200 Tuttlingen, Nussloch, German) at 3000 rpm and 35 °C, followed by washing, one step at a time, with 50 mL of THF and 50 mL of methanol under a stirring condition for 5 min. The washed Zn5 was then vacuum filtered (using a filtration system equipped with an A-32, EYELA water aspirator) after each washing, and it was dried at 65 °C for 12 h in an INB 400 Memmert oven, prior to use in subsequent runs.

### 2.4 Statistical Analysis

FAME yields were analyzed statistically by one-way analysis of variance performed using the PASW Statistics 18 program; P-values less than 0.05 were considered to indicate significant differences.

## 3 Results and Discussion

The as-prepared Zn5 material has similar characteristics to that of previous works,^8^ evidenced by all experiment results (Figure 1).

From Figure 1a., an IR strong band at 1370 cm^–1^ is attributed to the NO_2_ stretching mode of the free nitrate ion in the Zn5 material. The bands were in the 1000–1500 cm^–1^ characteristic range for nitrate stretching. The fingerprint region shows other nitrate absorption bands appearing at 722 cm^–1^ of symmetric deformation and near 829 cm^–1^ of asymmetric deformation. A very broad peak at 3000 to 3750 cm^–1^ corresponds to the hydroxyl group and perhaps is attributed to interactions among hydroxyl layers, water molecules, and nitrate groups. The Raman signal at 1051 cm^–1^ is due to N–O stretching for the free nitrate group^16^ in the Zn5 structure, as shown in Figure 1a. The diffraction peaks at 2-theta = 9.1, 18.2, 27.0, and 34.4° are typical characteristics of the Zn_5_(OH)_8_(NO_3_)_2_ phase according to the PDF 01-072-0627 database,^8^ as shown in Figure 1c. Next, methanolysis of various plant oil feedstock was conducted at 100 °C for 2 h by using a methanol-to-oil ratio of 30:1, similar to the previously reported conditions in a previous work.^8^ The screen test including XRD profiles in Figure 1d is indicative of the stability of Zn5 in the methanolysis reaction media. No crystalline phase changes were observed.

### 3.1 Methanolysis of Various Oil Feedstocks

The FAME yields obtained (Table 1.) suggested that Zn5 may not be an ideal material to promote high FAME yields from the plant oils under the studied conditions. On the other hand, by testing with various FFA feedstocks, stearic acid can be converted to FAME at the highest yields (Table 1). The FAME yields produced from PO or soybean oil, as well as lauric acid, are much lower than that obtained in previous works,^6^ which could be attributed to a different reactor setup (alcohol or stirring unit used).

Unlike plant oils, the diffraction patterns of spent Zn5 after methanolysis of FFAs (Figure 2a,b) indicate Zn5 reactivity resulting in the transformation of Zn5 to Zn–carboxylates accompanying the Zn5 minor phase. Despite the FAME yields of around 80% achieved from steric acid, Zn5 is nonrecyclable, and the highest FAME yield of around 60% obtained from olive oil is not ideal for any further recycling experiments. The results from Table 1 have brought our attention to explore the effects of acidity of oil feedstocks on the FAME yields. Thus, simulated acidic oil feedstocks were prepared for an additional FAME production screen test, and the results are given in Table 2.

The acidic oil feedstocks were consequently used for further reaction optimization and recycling experiments. The results shown in Figure 2c suggested that the Zn5 loadings of 3–5 wt % do give similar FAME yields of around 80%. A higher Zn loading of 7 wt % may have made mechanical agitation harder and led to slightly lower FAME yields. This could be because mass transfer between the oil, methanol, and catalyst phases was not as effective^14^ Therefore, a Zn5 loading of 3% wt was used in the following step. Notably, a mixture of palmitic acid/PO is denoted as *n*PA/PO; *n* = % wt of PA. By using Zn5, a high FAME yield of 80% or above can be obtained from methanolysis of PO feedstocks containing PA up to 10% (Figure 2d). Increasing the reaction temperature from 80 to 100 °C increased the FAME yield (Figure 2e), which is in line with previous work where the reactions were carried out in a closed system.^12^ However, a too high reaction temperature (120 °C) may result in rapid evaporation of the volatile reagent (methanol in this case). The high mobility of gaseous and liquid methanol may kinetically limit efficient methanolysis of the feedstock. Based on our statistical analysis and Figure 2c–f, the highest FAME yield of 97.5% was achievable by using 3% wt Zn5, a 30:1 MeOH to oil ratio at 100 °C for 2 h for the 3PA/PO acidic palm oils. Nevertheless, the spent Zn5 is a mixture of Zn5 and zinc palmitate (denoted as ZnP), as evidenced by the diffraction profile of Zn5/ZnP in Figure 3a,b, confirming the Zn5 reactivity toward palmitic acid, similar to the diffraction results (Figure 2b). Similarly, IR and Raman results in Figure 3c,d also confirmed the formation of the Zn5/ZnP composite. Although Zn5 resulted in high FAME yields in the methanolysis of acidic oils, it should not be considered a catalyst due to its instability in the reaction media.^15^

Reinoso et al. conducted a reaction between ZnCl_2_ with palmitic acid in a basic alcoholic solution, producing zinc palmitate, Zn(C_15_H_31_COO)_2_, which was an effective catalyst for FAME production from an acidic oil (10 wt % stearic acid/soybean oil). The authors proposed a mechanism involving the initial coordination of methanol, carboxylate shift, and the co-coordination of the triglyceride with an alkoxide-like moiety.^17^ In contrast, this study found that a Zn5/ZnP composite gave only around 50% FAME from 3PA/PO feedstocks. According to previous research and our findings, the composition of acidic feedstocks as well as Zn carboxylate materials may have a significant impact on FAME yields.

### 3.2 Recycling Experiments

No recycling studies were carried out on neat PO or palmitic acid by using Zn5, due to low FAME yields and Zn5 instability, respectively, as discussed earlier. Figure 3e displays the recyclability of Zn5/ZnP in the methanolysis of neat PO to produce high FAME yields up to four consecutive runs. The IR spectra in Figure 3c reflect the composition of Zn5, Zn5/ZnP, and the spent Zn5/ZnP from the conversion of PO to FAME. The nitrate content of Zn5/ZnP and spent Zn5/ZnP derived from the corresponding IR spectrum was found to be insignificantly different (Figure 3f), indicating the stability of Zn5/ZnP in the methanolysis reaction media, also supported by the data in Figure 3a–d. The Zn5/ZnP catalyst was found to be stable and able to maintain its activity. This study added more insight into the recyclability of Zn-carboxylates in FAME production. Zinc carboxylates (Zn-laurate, Zn-palmitate, and Zn stearate) were reported to give over 90% FAME yields from 10 wt % stearic acid/soybean oil.^18^ However, whether the carboxylates were effective in the subsequent runs needed to be tested. According to our findings, Zn5/ZnP (zinc carboxylate major phase) can be recycled in FAME production from oil feedstocks with low acidity. The lower Zn5/ZnP acidity, from 0.21 mmol of NaOH/g (cycle 1) to 0.12 mmol of NaOH/g (cycle 4), and methanolysis byproducts contaminating the solid active sites may have caused the reduced FAME yields in subsequent runs. Note that the basicity of Zn5 and Zn5/ZnP is negligibly different, in the range of 0.11 ± 0.04 mmol HCl/g, and the basicity of spent Zn5/ZnP is found to be in the same range. SEM images (Figure 4) show morphological changes, agglomerations, and pore filling, caused by methanolysis reactions and solid-state phase transformation. It is important to note that ZnP can blend into the same phase in the reaction media,^19^ but it returns to a solid form at room temperature, which can be easily recovered as the Zn5/ZnP composite after each cycle.

### 3.3 FAME Production Mechanisms

**Scheme 1:**
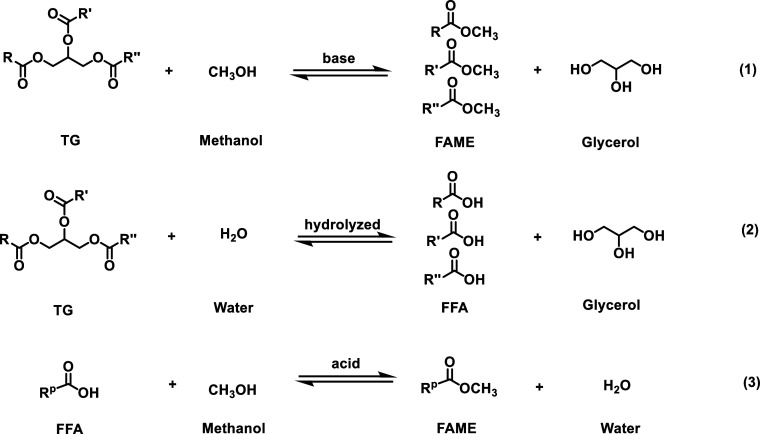
Major Reactions That Promote High FAME Yield in This Study R, R′, and R″ are alkyl fatty acid constituents, while R^p^ is alkyl fatty acid chain in palmitic acid.

Reactions involved in FAME productions from various feedstocks are described here, with methanol (MeOH), triglycerides (TG), and FFA serving as important reagents. The Zn5 material is recommended for FAME production from acidic oil feedstock, through simultaneous transesterification of TG, esterification of FFA, and TG hydrolysis reactions, all as shown in Scheme 1.

Note that Zn5 is a hydrate salt, which serves as the water source for TG hydrolysis, described in side reaction no. 3. The second cycle methanolysis of acidic oil by using Zn5 appeared to be ineffective for FAME production possibly due to soap formation taking place as well as the solid-state transformation of Zn5 to Zn5/ZnP. The results from this work suggested that Zn5/ZnP is an ideal material to promote high FAME yields from neat PO mainly through reaction no. 1–2 (Scheme 1). Additionally, reaction no. 2–3 can also occur, promoting relatively high FAME yields from neat PO compared to that reported in Table 1. The presence of acidic sites and base sites on the Zn5 and Zn5/ZnP surfaces, respectively, thus promoted effective FAME production from acidic oils and neat palm oils.

Table 3 summarizes the FAME yields obtained from methanolysis of various oil feedstocks using Zn5, Zn-carboxylates, and Zn5/ZnP. The acidity of oils feedstocks employed is also reported herein. The optimum FAME production conditions in this study are somewhat similar to those previously reported. Similar methanol-to-oil ratio (30:1) and high reaction temperatures (100 °C or above) were used for most feedstock, except for castor oil. Note that, regarding the recyclability of Zn5 reported by Reinoso’s group,^9^ using an oil feedstock with very low acidity, the researchers claimed that Zn5 remained stable across multiple tests (Table 3). The difference in FAME yields between this study and previous work could be attributed to different reaction conditions (e.g., alcohol used and internal pressure in the reactor) as well as differences in oil feedstock compositions (FFA and fatty acid chain length). For example, the reactivity of Zn5 in the conversion of soybean oil to FAME (Table 1) was found to be higher in a previous work^9^ probably due to the better mixing as they used a reactor equipped with a 4-angled blade stirrer. In another case, Cordeiro’s group^6^ used ethanol in FAME production in a closed system, resulting in varied internal pressure from the current work (methanolysis).

**Table 3:** FAME Yields Obtained from Methanolysis of Various Oil Feedstocks by Using Varying Zn5-Based Materials^a^

material	feedstock		reaction conditions					recyclability	FAME yield (%)
	name	acidity (mg NaOH/g)	catalyst (wt %)	MeOH/oil	temp (°C)	time (min)	pressure	(cycle)	
Zn5 (this work)	palm oil	0.1	3	30:1	100	120	h		22.0
Zn5^6^	palm oil		6	48:1	140	120			95.7
	lauric acid		6	6:1	140	120			96.2
Zn5^7^	castor oil		5	29:1	60	180		4	1st: 21.0
									2nd: 19.0
									3rd: 17.0
									4th: 16.0
									20.0
Zn5^9^	soybean oil		3	30:1	140	120		3	1st: 82.4
									2nd: 61.7
									3rd: 60.5
									76.8
Zn5^8^	soybean oil		10	30:1	140	120			98.9
	10% oleic acid/soybean oil		3						97.8
	3% palmitic acid/palm oil	4.58	3	30:1	100	120	h		97.5
Zn5 (this work)	5% palmitic acid/palm oil	7.41							92.8
	10% palmitic acid/palm oil	14.6							84.4
Zn5/ZnP (this work)	3% palmitic acid/palm oil	4.58	3	30:1	100	120			1st: 50.7
									2nd: 30.4
Zn5/ZnP (this work)	palm oil	0.1	3	30:1	100	120		4	1st: 97.0
									2nd: 91.7
									3rd: 77.2
									4th: 70.7
zinc palmitate^18^	soybean oil		3	30:1	140	120		3	1st: 73.3
									2nd: 66.1
									3rd: 65.5
									92.5
zinc acetate	soybean oil^18^		3	30:1	140	120			88.3
zinc laurate	soybean oil^18^							3	1st: 74.1
									2nd: 66.3
									3rd: 65.5
									91.3
zinc stearate								3	1st: 71.2
									2nd: 64.2
									3rd: 65.0
									86.0
zinc oleate									83.4
zinc oleate	oleic acid^18^		6	30:1	100	120		5	1st: 62.5
									2nd: 62.6
									3rd: 62.4
									4th: 62.5
									5th: 62.0
zinc oleate^21^	soybean oil								93.9
	10% oleic acid/soybean oil		3	30:1	140	120			75.9
	22% oleic acid/soybean oil								82.6

a The pressure description, ‘h’, represents that the reaction was conducted at 1.125 atm approximately, estimated from the theoretical methanol vapor pressure at 100 °C^20^ added with the atmospheric pressure.

## 4 Conclusions

According to our findings, Zn5 promoted high FAME yields from acidic oil feedstocks, but it is unstable and nonrecyclable. The Zn5/ZnP composites are not suitable for FAME production from acidic oils. However, if low-acidity oil feedstocks were used, the composites would be effective and recyclable catalysts. The effects of the alkyl chain length in Zn-carboxylate in the Zn-carboxylate/Zn5 composites on FAME yields produced from various oil feedstocks and any metal leaching in FAME should be further investigated. Such systematic investigation could provide valuable insights into optimizing the production of FAME from different oil feedstocks using Zn-carboxylate/Zn5 composites and help identify the most efficient catalyst for FAME production. It appears that appropriate matching between oil feedstocks and the acidity/basicity of Zn5-based materials, including reaction conditions, significantly influences FAME yields.
